# Insights into the effects of geographical sourcing area on nutrient composition and sensory attributes of nine edible insects

**DOI:** 10.1038/s41598-025-90659-z

**Published:** 2025-04-04

**Authors:** Jackson Ishara, Rehema Matendo, Jeremiah Ng’ang’a, Saliou Niassy, Karume Katcho, John Kinyuru

**Affiliations:** 1 Department of Food Science and Technology, Université Evangélique en Afrique, P.O. Box 3323, Bukavu, Democratic Republic of the Congo; 2 Department of Food Science and Technology, Jomo Kenyatta University of Agriculture and Technology, P.O. Box 62000-00200, Nairobi, Kenya; 3 Faculty of Agriculture and Environmental Sciences, Université de Kaziba, P.O. Box 2106, Bukavu, Democratic Republic of the Congo; 4 Department of Environmental and Agronomic Sciences, Université Officielle de Bukavu, P.O. Box 570, Bukavu, Democratic Republic of the Congo; 5 Inter-African Phytosanitary Council of African Union (AU-IAPSC), P.O Box 4170, Yaoundé, Cameroon; 6 Department of Zoology and Entomology, Faculty of Natural and Agricultural Sciences, University of Pretoria, Hatfield, Gauteng, Pretoria South Africa; 7 Faculty of Agriculture and Environmental Sciences, Université Evangélique en Afrique, 3323 Bukavu, Democratic Republic of the Congo; 8 Centre de Recherche en Géothermie, 327, Bukavu, Democratic Republic of the Congo

**Keywords:** Edible insects, Geographical sourcing area, Nutrient composition, Sensory attributes, Biochemistry, Biotechnology, Ecology

## Abstract

With the rapidly growing world population, climate change uncertainties and depletion of our natural resources, edible insects are seen as sustainable and viable bioresources for food and feed to tackle global food and nutritional security issues, for their nutritional value, taste, and environmental sustainability. In this study, we investigated the effect of geographical distribution on the nutrient composition and sensory attributes of commonly consumed edible insects, namely *Acheta domesticus*, *Apis mellifera*, *Gnathocera trivittata*, *Gryllotalpa africana*, *Imbrasia oyemensis*, *Locusta migratoria*, *Macrotermes subhylanus*, *Nomadacris septemfasciata*, and *Rhyncophorus phoenicis* collected from six different geographical areas namely Fizi, Kabare, Kalehe, Idjwi, Mwenge and Walungu, in the Eastern D. R. Congo. Depending on edible insect species, geographical sourcing area affected significantly macronutrient composition and mineral profile as well as sensory attributes of investigated commonly edible insects. A principal component analysis (PCA-Biplot) indicated that the two axes accounted for up to 97.7% of the observed variability in the nutrient composition and sensory attributes of commonly consumed edible insects sourced from different geographical area. Visualized results after cluster analysis using non-metric multidimensional scaling (NMDS) indicated that the geographical sourcing area has substantial and significant effect on the nutrient composition as well as sensory attributes of the studied commonly edible with a stress value of 0.185.

**Supplementary Information:**

The online version contains supplementary material available at 10.1038/s41598-025-90659-z.

## Introduction

Edible insects are seen as sustainable and viable bioresources for food and feed to address global food and nutrition security issues^1,2^ linked to rapid global population growth^3^, climate change uncertainties^4^ and the depletion of our natural resources^5^. There are an estimated 5.5 million insect species worldwide, of which only about 1 million have been described^6^, and over 2,100 are consumed, mainly in tropical countries, and are categorized as beetles (Coleoptera, 31%), caterpillars (Lepidoptera, 18%), bees, wasps and ants (Hymenoptera, 14%), locusts, grasshoppers, crickets and crickets (Orthoptera, 13%), cicadas, leafhoppers, grasshoppers, mealybugs and bedbugs (Hemiptera, 10%), termites (Isoptera, 3%), dragonflies (Odonata, 3%), flies (Diptera, 2%) and 5% other orders being the most widely consumed insect groups^7^.

Several studies have paid attention to nutritional composition of edible insects reporting protein content ranging from 6.25 to 80.26% on dry matter, fat content from 2.2 to 43%, 1.91–9.2% (moisture content), 1.2-11.38% (ash content) and a rang from 1.01 to 6.8% for fiber content^8–13^. Additionally, as edible insects emerges as a mainstream food source, their acceptance and adoption largely depend on consumer attitudes and perceptions^14^, of which sensory attributes including taste, texture, aroma, and appearance play a crucial role^15^. As our understanding of edible insects continues to grow, efforts are being made to develop innovative preparation techniques and recipes that optimize their sensory appeal^16^. With their diverse flavors, unique textures, intriguing aromas, and visually captivating appearances^17^.

However, their nutritional content and sensory attributes are subjected to biotic factors including diet, harvesting time and gender, age, and abiotic factors such as temperature, humidity and light^18^, in addition to processing methods^19^ and preservation techniques^13^ as well as applied analytic method^20^. A number of studies have shown the importance of feeding behavior on nutritional status of edible insects^21–23^, so it is possible to diversify diet in order to meet specific needs.

Although edible insects have gained particular attention, only 10% are currently described, and less so in regard to the effects of geographical sourcing area on their nutritional composition and sensory attributes, despite the fact that the latter are largely depending on biogeochemical conditions. Located in the Eastern of the Democratic Republic of Congo, Fizi, Idjwi, Kabare, Kalehe, Mwenga and Walungu are relatively six territories well known for the widespread of anthropo-entomophagy practices, with diverse agro-ecological conditions^24^. While the Fizi, Walungu, kabare and Mwenga territories are characterized by acrisols, cambisols, ferralsols and nitisols, the Kalehe and idjwi territories are characterized by haplic acrisols, dystric cambisols and haplic nitisols, humid ferralsols and gleyic solonchaks with different climatic conditions likely to affect not only land/plant cover but also the nutrient composition and sensory attributes of edible insects^24^. Considering the diversity in term of soil composition, climate, temperature, humidity, rainfall among other biogeochemical conditions, this study therefore aimed at investigating the effect of geographical sourcing area on the nutritional composition and sensory attributes of commonly edible insects in Eastern Democratic Republic of Congo.

## Results

### Influence of geographical sourcing area on nutrient composition

The geographical sourcing effect on macronutrient composition of commonly consumed edible insects in Eastern D. R. Congo is depicted in Table 1. Although the geographical sourcing area did not affect significantly (*p* > 0.05) the protein content for *Acheta domesticus*, *Apis mellifera*, *Gnathocera trivittata*, *Grillotalpa africana* and *Nomadacris septemfasciata*, a significant effect (*p* < 0.05) was observed for *Imbrasia oyemensis*, *Locusta migratoria*, *Macrotermes subhyalinus* and *Rhynchophorus phoenicis*. Fat content varied significantly for all edible insects except for *G. trivittata*. Ash content varied significantly (*p* < 0.05) for *A. domesticus*, *A. mellifera* and *M. subhyalinus*, but not for *G. trivittata*, *G. africana*, *I. oyemensis*, *L. migratoria*, *N. septemfasciata* and *R. phoenicis*. A significant variation (*p* < 0.05) in moisture content was observed for all studied edible insects except for *R. phoenicis*.

*Acheta domesticus* macronutrient composition varied significantly (*p* < 0.05) due to geographical sourcing area, except its protein content (*p* > 0.05), which ranged between 35.25 and 38.16 g/100 g, with fat content ranging between 20.99 and 24 g/100 g, ash content 4.90 to 11.83 g/100 g and moisture content 57.23 to 66.25 g/100 g. As for *A. mellifera*, its protein content ranged between 19 and 20.08 g/100 g, fat (22.27–26.29 g/100 g), ash (4.41–8.79 g/100 g) and between 70.06 and 78.84 g/100 g for moisture content.

*Gnathocera trivittata* had a protein content ranging between 34.40 and 36.13 g/100 g, fat content (16.90 to 17.78 g/100 g), ash (4.94 to 6.57 g/100 g), and moisture content (56.40 to 62.21 g/100 g). The protein, fat, ash as well as moisture content of *G. africana* ranged between 30.70 and 32.22 g/100 g, 21.23–26.73 g/100 g, 4.45–5.26 g/100 g, and 63.66–79.72 g/100 g respectively. *Imbrasia oyemensis* was found to be nutritionally potential with 31.18–56.68 g/100 g (protein content), 15.68–27.95 g/100 g (fat), 5.92–6.93 g/100 g (ash), 11.94–28.57 g/100 g (CHO), and 66.58–79.05 g/100 g for moisture. Similarly for *L. migratoria*, with protein, fat, ash, and moisture contents ranging from 29.63 to 35.25 g/100 g, 16.95–23.10 g/100 g, 4.54–5.67 g/100 g and 59.82–77.34 g/100 g respectively. Finally, *R. phoenicis*, with a protein content of 31.55–39.19 g/100 g, fat content (25.80–30.60 g/100 g), ash content (6.92–7.02 g/100 g) and moisture content (68.14–68.84 g/100 g).

**Table 1:** Geographical sourcing effect on macronutrient composition of commonly consumed edible insects in Eastern D. R. Congo.

Insect species/territory	Protein (g/100 g)	Fat (g/100 g)	Ash (g/100 g)	MC (%)
*Apis mellifera*				
Idjwi	19.07 ± 1.10a	24.49 ± 0.87ab	8.79 ± 3.92a	70.06 ± 1.02b
Kabare	20.08 ± 0.49a	24.38 ± 1.16ab	4.72 ± 0.36b	71.23 ± 2.08b
Kalehe	19.66 ± 2.51a	26.29 ± 1.72a	4.89 ± 0.31b	78.84 ± 2.11a
Walungu	19.82 ± 1.02a	22.27 ± 0.91b	4.41 ± 0.59b	71.59 ± 1.72b
p-value	0.793	0.023	0.043	< 0.001
*Gnathocera trivittata*				
Kabare	34.40 ± 0.66a	17.78 ± 0.29a	4.94 ± 0.61a	62.21 ± 1.68a
Walungu	36.13 ± 2.92a	16.90 ± 0.7a	6.57 ± 0.86a	56.40 ± 1.78b
p-value	0.305	0.115	0.055	0.015
*Grillotalpa africana*				
Kabare	30.70 ± 1.10a	26.73 ± 0.63a	5.26 ± 0.46a	63.66 ± 0.35b
Walungu	32.22 ± 1.25a	21.23 ± 0.96b	4.45 ± 0.33a	79.72 ± 1.45a
p-value	0.136	< 0.001	0.067	< 0.001
*Imbrasia oyemensis*				
Kalehe	31.18 ± 1.09b	27.95 ± 1.41a	6.93 ± 0.18a	79.05 ± 2.30a
Mwenga	56.68 ± 1.20a	15.68 ± 1.00b	5.92 ± 1.01a	66.58 ± 1.07b
p-value	< 0.001	< 0.001	0.166	< 0.001
*Locusta migratoria*				
Idjwi	29.63 ± 0.90b	21.97 ± 0.15ab	5.17 ± 1.02a	69.03 ± 1.60b
Kabare	35.25 ± 1.53a	21.14 ± 0.25ab	5.67 ± 0.50a	59.82 ± 0.22c
Kalehe	30.08 ± 0.36b	16.95 ± 0.73c	5.56 ± 0.68a	77.34 ± 2.32a
Walungu	30.22 ± 1.08b	23.10 ± 1.51a	4.54 ± 1.17a	69.22 ± 1.46b
p-value	< 0.001	< 0.001	0.437	< 0.001
*Macrotermes subhyalinus*				
Fizi	29.00 ± 1.06a	27.10 ± 0.93a	8.93 ± 0.68a	45.47 ± 0.55c
Kabare	25.77 ± 2.05b	26.34 ± 1.59a	5.01 ± 0.39b	70.33 ± 0.59b
Walungu	27.88 ± 2.19ab	24.90 ± 1.23b	4.55 ± 0.52b	77.27 ± 2.85a
p-value	0.011	0.021	< 0.001	< 0.001
*Nomadacris septemfasciata*				
Kabare	24.54 ± 1.57a	38.28 ± 1.53a	5.50 ± 0.82a	56.43 ± 1.16b
Walungu	30.61 ± 4.86a	11.97 ± 1.29b	4.30 ± 0.16a	66.31 ± 0.84a
p-value	0.073	< 0.001	0.069	< 0.001
*Rhynchophorus phoenicis*				
Fizi	31.55 ± 0.56b	25.80 ± 1.31b	6.92 ± 1.62a	68.84 ± 0.56a
Idjwi	39.19 ± 3.44a	30.60 ± 1.05a	7.02 ± 0.69a	68.14 ± 0.32a
p-value	0.011	0.008	0.924	0.133
*Acheta domesticus*				
Fizi	37.58 ± 1.56a	24.00 ± 2.00a	11.83 ± 0.61a	57.23 ± 1.57b
Kabare	37.19 ± 4.58a	20.99 ± 0.79b	5.09 ± 0.16b	65.50 ± 1.80a
Mwenga	35.25 ± 0.92a	22.70 ± 1.00ab	4.90 ± 1.00b	57.74 ± 1.02b
Walungu	38.18 ± 4.33a	21.43 ± 0.78b	4.94 ± 0.61b	66.25 ± 1.21a
p-value	0.628	0.028	< 0.001	< 0.001

Mean values (*n* = 3) ± SE. All values except moisture are expressed on dry weight.

Values in the same column with the same following letter do not significantly differ (*p* < 0.05).

*MC* Moisture content.

### Geographical sourcing effect on mineral profile of commonly consumed edible insects in Eastern D. R. Congo

Table 2 presents the geographical sourcing area effect on the potassium, sodium, magnesium, iron, calcium and zinc content of *A. domesticus*, *A. mellifera*, *G. trivittata*, *G. africana*, *I. oyemensis*, *L. migratoria*, *M. subhyalinus*, *N. septemfasciata* and *R. phoenicis*, commonly edible insects in Eastern D. R. Congo. Although the geographical sourcing area affected significantly (*p* < 0.05) the potassium, sodium, iron, calcium and zinc content in studied edible insects, the magnesium content was not significantly (*p* > 0.05) affected for *A. domesticus* and *A. mellifera*. As for *A. mellifera*, its potassium, sodium, iron and calcium content varied significantly (*p* < 0.05), except for its magnesium and zinc content. Potassium, sodium, magnesium, iron and calcium content varied significantly (*p* < 0.05) in *G. trivittata*, except for its zinc content (*p* > 0.05).

Geographical sourcing area affected significantly (*p* < 0.05) potassium, sodium, magnesium, calcium and zinc content for *G. africana*, except its iron content. In contrast to previous species, geographical sourcing area significantly (*p* < 0.05) affected some mineral (potassium, sodium and calcium) content in *I. oyemensis*, while other mineral (magnesium, iron and zinc) content was not significantly (*p* > 0.05) affected. All assessed mineral varied significantly (*p* < 0.05) in regards to geographic sourcing area for *L. migratoria*. Geographical sourcing area affected significantly (*p* < 0.05) all assessed mineral, with the exception of iron for *M. subhyalinus* and sodium for *R. phoenicis*. As for *N. septemfasciata*, its potassium, magnesium, iron and calcium content varied significantly (*p* < 0.05) in respect to geographical sourcing area, except for calcium and zinc.

The potassium, sodium, magnesium, iron, calcium and zinc content of *A. domesticus* ranged between 67.27 and 148.25 mg/100 g, 146 and 161.23 mg/100 g, 40.90 and 59.60 mg/100 g, 4.1 and 6.6 mg/100 g. 87 and 144.27 mg/100 g and 12.2-15.85 mg/100 g respectively. As for *A. mellifera*, its potassium content varied between 74.79 and 101.7 mg/100 g, sodium (143.5–158.67 mg/100 g), magnesium (45.33–51.72 mg/100 g), iron (4.87–9.24 mg/100 g), calcium (124–147.4 mg/100 g) and zinc (14.27–15 mg/100 g). For *G. trivittata*, its potassium content varied between 19.54 and 105.38 mg/100 g, 144.23–161.3 mg/100 g (sodium), 32.4–54.77 mg/100 g (magnesium), 6.5–8.8 mg/100 g (iron), 128.5–148.33 mg/100 g (calcium) and 14.4–15.83 mg/100 g (zinc). The *G. africana* species was found to be rich in potassium (46.91–81.3 mg/100 g), sodium (141.47–168 mg/100 g), magnesium (24.3–42.17 mg/100 g), iron (5.05–5.53 mg/100 g), calcium (129.5–162 mg/100 g) and zinc (15.1–16.3 mg/100 g).

For *I. oyemensis*, potassium, sodium, magnesium, iron, calcium and zinc contents ranged between 56.43 and 193.16 mg/100 g, 154.44–159.23 mg/100 g, 60.57–66.96 mg/100 g, 7.47–7.57 mg/100 g, 102–107.33 mg/100 g, 12.38–14.36 mg/100 g respectively. With a potassium content of 73.43–108.05 mg/100 g, sodium (154–162.9 mg/100 g), magnesium (44.83–63.43 mg/100 g), iron (4.15–7.17 mg/100 g), calcium (122.67–153 mg/100 g) and zinc (13.1–17.13 mg/100 g), *L. migratoria* was found to be a good source of minerals. *Macrotermes subhyalinus* is also a good source of minerals, with 160.43–520.44 mg/100 g (potassium), 157.67–164.33 mg/100 g (sodium), 20.6–67.87 mg/100 g (magnesium), 5.2–5.9 mg/100 g (iron), 97.33–139.1 mg/100 g (calcium) and 15.1–17.8 mg/100 g (zinc). While for *N. septemfasciata* the potassium, sodium, magnesium, iron, calcium and zinc content varied respectively between 54.37 and 116.17 mg/100 g, 162.07–162.7 mg/100 g, 29.8–56.27 mg/100 g, 6.8–7.79 mg/100 g, 153–160.5 mg/100 g and 13.03–13. 2 mg/100 g, the potassium, sodium, magnesium, iron, calcium and zinc content of *R. phoenicis* ranged from 27.65 to 28.93 mg/100 g, 169.33–170.67 mg/100 g, 32.3–58.93 mg/100 g, 5.43–8.57 mg/100 g, 98.97–176.05 mg/100 g and 13–18.3 mg/100 g respectively.

**Table 2:** Geographical sourcing effect on mineral profile (mg/100 g) of commonly consumed edible insects in Eastern D. R. Congo.

Insect species/Territory	Potassium	Sodium	Magnesium	Iron	Calcium	Zinc
*Apis mellifera*						
Idjwi	98.37 ± 1.37ab	143.50 ± 1.42d	49.27 ± 6.33a	9.24 ± 0.06a	147.40 ± 0.56a	14.68 ± 0.63a
Kabare	94.20 ± 3.13b	154.70 ± 0.40b	50.10 ± 2.90a	6.60 ± 0.20b	124.00 ± 1.00b	15.00 ± 0.60a
Kalehe	101.70 ± 3.54a	152.23 ± 1.24c	51.72 ± 3.97a	6.50 ± 0.85b	124.33 ± 2.52b	14.53 ± 0.72a
Walungu	74.79 ± 1.94c	158.67 ± 1.53a	45.33 ± 0.78a	4.87 ± 0.21c	126.33 ± 1.53b	14.27 ± 0.47a
p-value	< 0.001	< 0.001	0.321	< 0.001	< 0.001	0.399
*Gnathocera trivittata*						
Kabare	19.54 ± 0.63a	161.30 ± 0.30a	32.40 ± 2.50b	8.80 ± 0.10a	128.50 ± 7.50b	14.40 ± 0.50a
Walungu	105.38 ± 1.20b	144.23 ± 1.07b	54.77 ± 1.30a	6.50 ± 0.53b	148.33 ± 3.21a	15.83 ± 0.90a
p-value	< 0.001	< 0.001	< 0.001	< 0.001	0.013	0.496
*Grillotalpa africana*						
Kabare	46.91 ± 1.41b	168.00 ± 0.20a	24.30 ± 0.34b	5.09 ± 0.21a	129.50 ± 0.50b	15.10 ± 0.40a
Walungu	81.03 ± 1.03a	141.47 ± 0.72b	42.17 ± 1.21a	5.53 ± 0.38a	162.00 ± 2.00a	16.30 ± 0.44a
p-value	< 0.001	< 0.001	< 0.001	0.373	< 0.001	0.101
*Imbrasia oyemensis*						
Kalehe	193.16 ± 2.15a	159.23 ± 0.88a	66.96 ± 9.08a	7.47 ± 0.49a	107.33 ± 1.53a	14.36 ± 1.63a
Mwenga	56.43 ± 1.19b	154.44 ± 1.16b	60.57 ± 0.86a	7.57 ± 0.90a	102.00 ± 1.00b	12.38 ± 1.22a
p-value	< 0.001	0.004	0.306	0.643	0.007	0.158
*Locusta migratoria*						
Idjwi	73.43 ± 0.97d	157.18 ± 1.02b	56.00 ± 4.40b	4.15 ± 0.05b	131.49 ± 0.50b	13.31 ± 0.38b
Kabare	77.75 ± 1.26c	154.00 ± 0.56c	51.40 ± 1.10bc	6.00 ± 0.10a	153.00 ± 4.00a	13.10 ± 0.80b
Kalehe	87.40 ± 2.17b	157.54 ± 1.51b	63.43 ± 6.26a	5.93 ± 0.40a	125.33 ± 1.53c	13.19 ± 1.37b
Walungu	108.05 ± 1.27a	162.90 ± 0.66a	44.83 ± 0.60c	7.17 ± 0.64a	122.67 ± 2.52c	17.13 ± 0.35a
p-value	< 0.001	< 0.001	0.002	0.003	< 0.001	< 0.001
*Macrotermes subhyalinus*						
Fizi	480.47 ± 0.39b	157.67 ± 0.58b	28.73 ± 1.12b	5.57 ± 0.42a	139.10 ± 0.85a	17.57 ± 0.75a
Kabare	160.43 ± 0.03c	163.90 ± 0.40a	20.60 ± 1.60c	5.90 ± 0.23a	135.50 ± 0.50b	17.80 ± 0.40a
Walungu	520.44 ± 19.77a	164.33 ± 0.68a	67.87 ± 0.97a	5.20 ± 0.20a	97.33 ± 1.53c	15.10 ± 0.36b
p-value	< 0.001	< 0.001	< 0.001	0.123	< 0.001	0.009
*Nomadacris septemfasciata*						
Kabare	54.37 ± 2.15b	162.70 ± 1.40a	29.80 ± 6.50b	6.80 ± 0.20a	160.50 ± 2.50a	13.20 ± 0.30a
Walungu	116.71 ± 0.77a	162.07 ± 1.69a	56.27 ± 1.16a	7.79 ± 0.36a	153.00 ± 1.00b	13.03 ± 0.70a
p-value	< 0.001	0.621	0.002	0.101	0.009	0.865
*Rhynchophorus phoenicis*						
Fizi	28.93 ± 0.73a	169.33 ± 0.58a	32.30 ± 0.30b	8.57 ± 0.42a	176.05 ± 0.45a	18.30 ± 0.60a
Idjwi	27.65 ± 0.76a	170.67 ± 1.35a	58.93 ± 4.46a	5.43 ± 0.81b	98.97 ± 0.35b	13.00 ± 2.05b
p-value	0.101	0.349	< 0.001	< 0.001	< 0.001	0.029
*Acheta domesticus*						
Fizi	148.25 ± 0.49a	160.67 ± 0,81a	47.60 ± 0.60a	6.60 ± 0.20a	144.27 ± 4.56a	15.85 ± 0.35a
Kabare	144.56 ± 1.56a	146.00 ± 1.00c	46.60 ± 9.00a	6.27 ± 0.23a	143.00 ± 4.00a	14.50 ± 0.10b
Mwenga	67.27 ± 3.88c	157.40 ± 1.00b	59.60 ± 20.00a	4.10 ± 0.10c	131.00 ± 1.00b	12.20 ± 1.00c
Walungu	110.93 ± 0.67b	161.23 ± 0.93a	40.90 ± 0.56a	5.87 ± 0.29b	87.00 ± 1.65c	15.40 ± 0.36ab
p-value	< 0.001	< 0.001	0.280	< 0.001	< 0.001	< 0.001

Mean values (*n* = 3) ± SE on wet basis. Values in the same column with the same following letter do not significantly differ (*p* < 0.05).

### Geographical sourcing effect on sensory attributes of commonly consumed edible insects in Eastern D. R. Congo

**Fig. 1:**
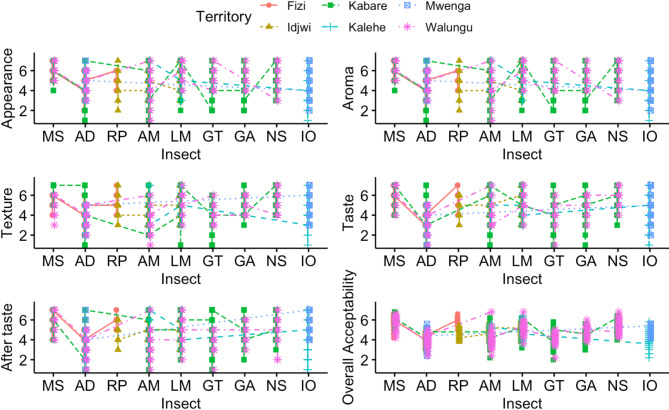
Geographical sourcing effect on sensory attributes of commonly consumed edible insects in Eastern D. R. Congo. MS: *M. subhyalinus*; AD: *A. domesticus*; RP: *R. phoenicis*; AM: *A. mellifera*; LM: *L. migratoria*; GT: *G. trivittata*; GA: *G. africana*; NS: *N. septemfasciata*; and IO: *I. oyemensis*.

The geographical sourcing area affected differently the sensory attributes namely appearance, aroma, texture, taste, after taste and overall acceptability of commonly edible insects (*A. domesticus*, *A. mellifera*, *G. trivittata*, *G. africana*, *I. oyemensis*, *L. migratoria*, *M. subhyalinus*, *N. septemfasciata* and *R. phoenicis*) in Eastern D. R. Congo as depicted in Table S3 and Fig. 1. The geographical sourcing area did not significantly (*p* > 0.05) affect the sensory attributes of *A. domesticus* except its aroma. On the other hand, it did affect significantly the sensory attributes of *A. mellifera*. While appearance, aroma and texture of *G. trivittata* varied significantly (*p* < 0.05) with geographical sourcing area, taste, after taste and overall acceptability did not. For *G. africana* and *R. phoenicis*, the geographical sourcing area significantly (*p* < 0.05) affected all sensory attributes. As for *I. oyemensis*, aroma, texture, after taste and overall acceptability varied significantly (*p* < 0.05) with geographical sourcing area, unlike appearance and taste. While the appearance, aroma, taste and after taste of *L. migratoria* varied significantly (*p* < 0.05) with geographical sourcing area, its texture and overall acceptability did not vary significantly (*p* > 0.05). The appearance, texture and taste of *M. subhyalinus* were significantly (*p* < 0.05) affected by geographical sourcing area, but its aroma, after taste and overall acceptability did not vary significantly (*p* > 0.05). An exception was noted for *N. septemfasciata*, all its sensory attributes did not vary significantly (*p* > 0.05) with geographical sourcing area except its taste (*p* < 0.05).

**Fig. 2:**
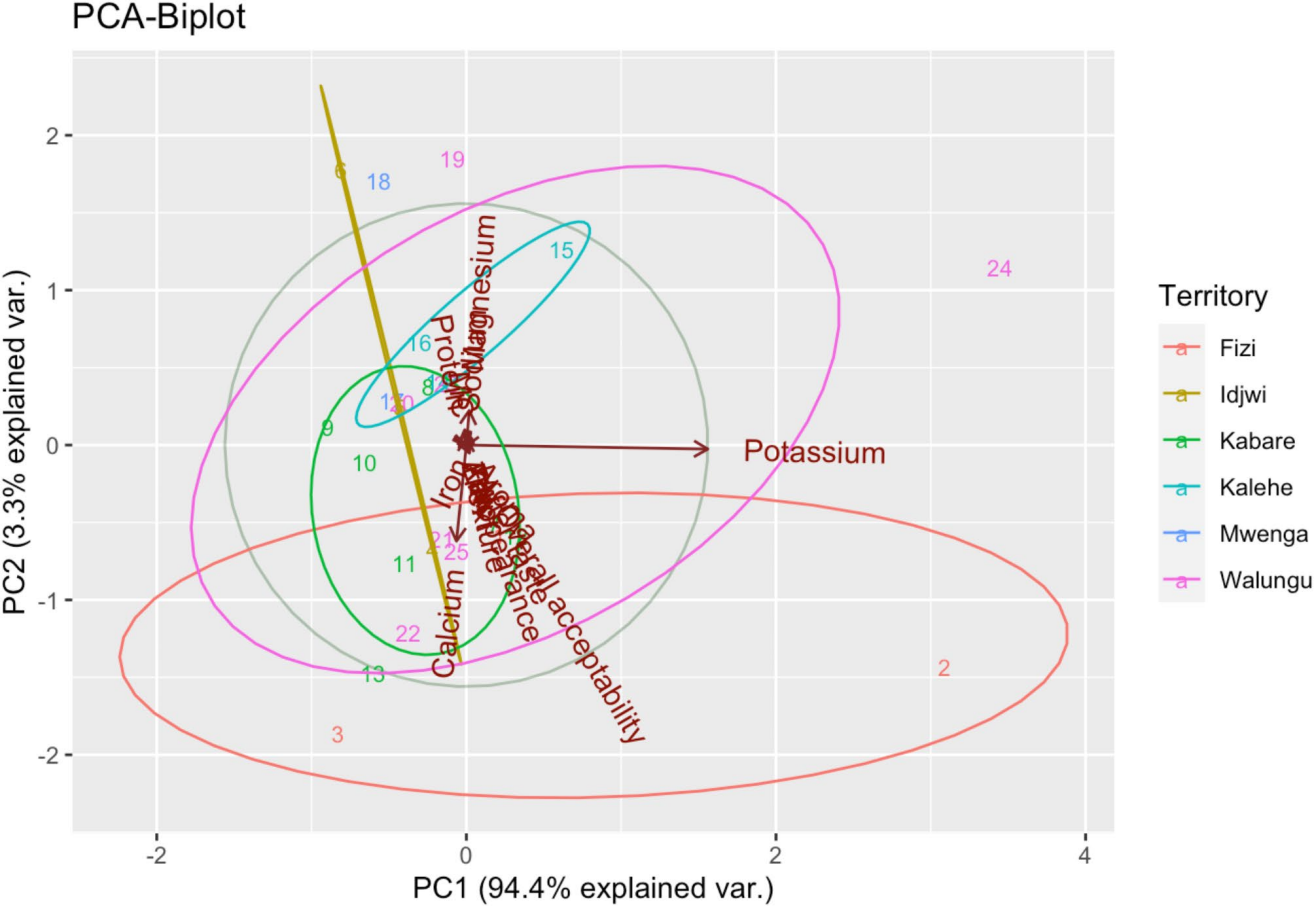
PCA-Biplot of nutrient composition and sensory attributes. (1) *A. domesticus*_Fizi; (2) *M. subhyalinus*_Fizi ; 3.*R. phoenicis*_Fizi; 4. *A. mellifera*_Idjwi; 5. *L. migratoria*_Idjwi; 6. *R. phoenicis*_Idjwi; 7. *A. domesticus*_Kabare 8. *A. mellifera*_Kabare; 9. *G. trivittata*_Kabare; 10. *G. africana*_Kabare; 11. *L. migratoria*_Kabare; 12. *M. subhyalinus*_Kabare; 13. *N. septemfasciata*_Kabare; 14. *A. mellifera*_Kalehe; 15. *I. oyemensis*_Kalehe; 16. *L. migratoria*_Kalehe; 17. *A. domesticus*_Mwenga; 18. *I. oyemensis*_Mwenga; 19. *A. domesticus*_Walungu; 20. *A. mellifera*_Walungu; 21. *G. trivittata*_Walungu; 22. *G. africana*_Walungu; 23. *L. migratoria*_Walungu; 24. *M. subhyalinus*_Walungu; 25. *N. septemfasciata*_Walungu.

**Fig. 3:**
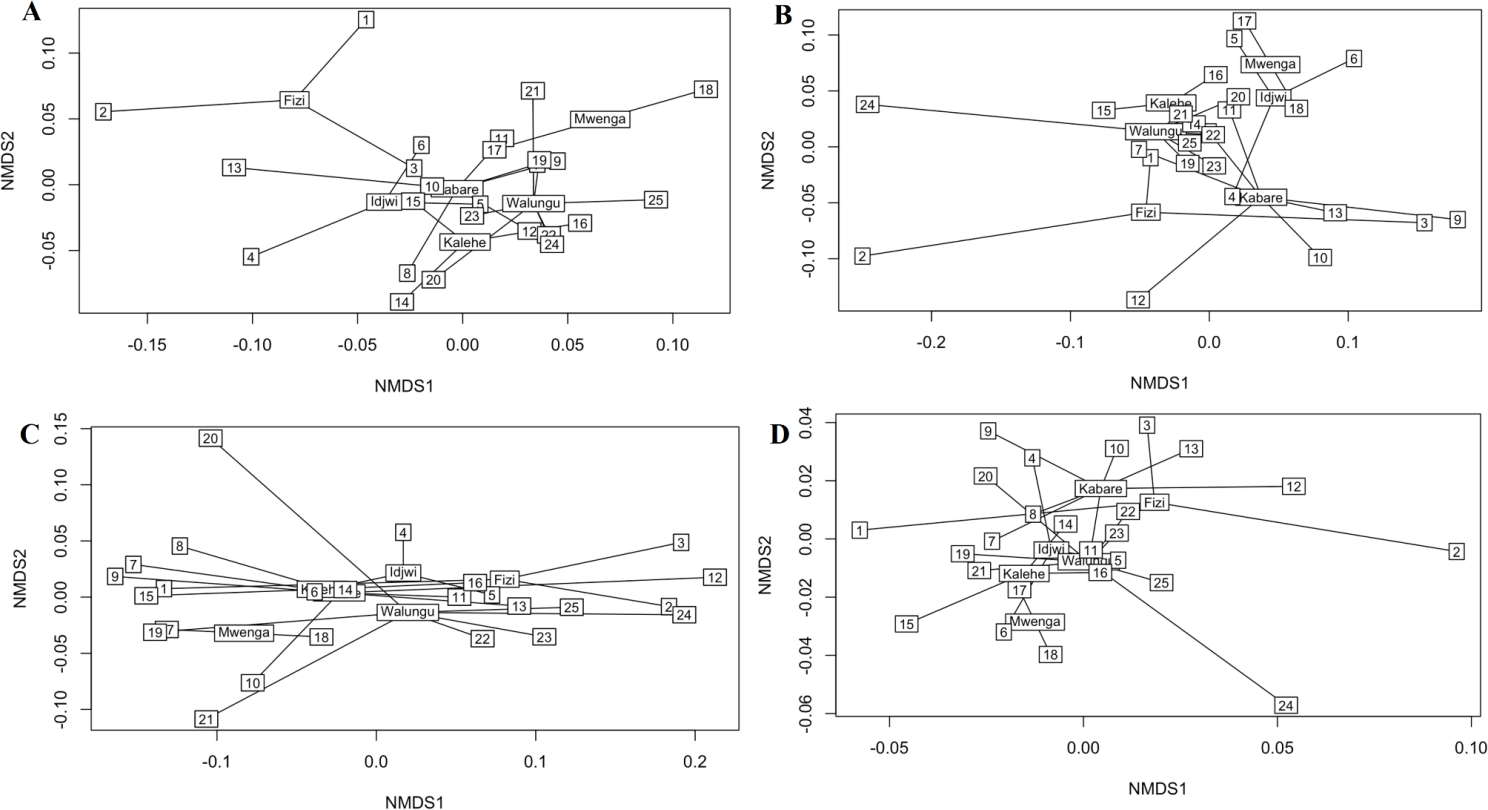
Cluster analysis using non-metric multidimensional scaling (NMDS) to determine the extent to which geographical sourcing area influenced edible insects. A: macronutrient composition (stress value 0.138 and *p* = 0.043), B: mineral profile (stress value 0.107 and *p* = 0.045), C: sensory attributes (stress value 0.095 and *p* = 0.039), and D: all parameters combined (stress value 0.185 and *p* = 0.024), The distance between different points on the plot reflects their similarity level: the more similar the composition of the samples, the smaller the distance between the points. (1) *A. domesticus*_Fizi; (2) *M. subhyalinus*_Fizi ; 3.*R. phoenicis*_Fizi; 4. *A. mellifera*_Idjwi; 5. *L. migratoria*_Idjwi; 6. *R. phoenicis*_Idjwi; 7. *A. domesticus*_Kabare 8. *A. mellifera*_Kabare; 9. *G. trivittata*_Kabare; 10. *G. africana*_Kabare; 11. *L. migratoria*_Kabare; 12. *M. subhyalinus*_Kabare; 13. *N. septemfasciata*_Kabare; 14. *A. mellifera*_Kalehe; 15. *I. oyemensis*_Kalehe; 16. *L. migratoria*_Kalehe; 17. *A. domesticus*_Mwenga; 18. *I. oyemensis*_Mwenga; 19. *A. domesticus*_Walungu; 20. *A. mellifera*_Walungu; 21. *G. trivittata*_Walungu; 22. *G. africana*_Walungu; 23. *L. migratoria*_Walungu; 24. *M. subhyalinus*_Walungu; 25. *N. septemfasciata*_Walungu.

In Fig. 2, results on Principal component analysis (PCA-Biplot) indicated that the two axes accounted for up to 97.7% of the observed variability in the nutrient composition and sensory attributes of commonly consumed edible insects sourced from different geographical area in Eastern D. R. Congo. The first and second axes accounted for 94.4% and 3.3% of variability, respectively. Visualized results after cluster analysis using non-metric multidimensional scaling (NMDS) indicated that the geographical sourcing area has substantial and significant effect on the nutrient composition as well as sensory attributes of commonly edible in the Eastern D. R. Congo with stress value of 0.185 and *p* = 0.043 as depicted in Fig. 3D. In the macronutrient composition NMDS plot (Fig. 3A), mineral NMDS plot (Fig. 3B) as well as sensory NMDS plot (Fig. 3C), the edible insects are distant from each other indicating the influence of geographical sourcing area with stress value 0.138 and *p* = 0.043 (macronutrient composition), stress value 0.107 and *p* = 0.045 (mineral profile), as well as stress value 0.095 and *p* = 0.039 (sensory attributes).

## Discussion

The macronutrient composition and sensory quality of edible insects depend on several aspects, including edible insect species and their development stage^25^, feeding habit^26^, processing methods^27^ and geographical sourcing area^28^, as well as measurement methods^29^. The significant geographical sourcing area effect on both nutrient composition and sensory attributes revealed in this study affirms the findings of Romotowska et al.^30^, Joy et al.^31^ and Manditsera et al.^32^ who reported that soil type, geographical sourcing area and season can influence the chemical composition of vegetation, and consequently that of animals such as edible insects that feed on the latter^33^. It is therefore possible that the difference between soil and vegetation in the different samples sourcing territories influenced the nutrient composition and sensory attributes of the studied edible insects.

This difference could be attributed to the biogeochemistry diversity in the sourcing area as Fizi territory is dominated by acrisols and cambisols with humid wet and dry tropical climate type, Walungu territory is dominated by ferralsols, cambisols and nitisols with humid wet tropical climate type, but Kabare territory is dominated by ferralsols and nitisols with humid wet tropical climate type. While Mwenga territory is dominated by acrisols and cambisols with equatorial climate type, Kalehe territory is dominated by haplic acrisols, dystric cambisols and haplic nitisols and humid ferralsols with humid wet tropical climate type, and Idjwi territory is dominated by gleyic solonchaks, nitisols and humid ferralsols with humid wet tropical climate type^24^. This corroborates with the findings of Sokol et al.^34^, who reported that the difference in soil mineral composition is caused by differences in either the type or magnitude of the factors that influence soil nutrient composition, such as parent (rock) material, climate, soil particle size, pH, humus content, aeration, temperature, water content, root surface area and mycorrhizal development among others.

In this study, the geographical sourcing area affected significantly the fat, ash, moisture, potassium, sodium, magnesium, iron, calcium and zinc content of *A. domesticus*, confirming the findings of Bawa et al.^21^, who indicated that feeding habits had impacted significantly the protein, fat, ash, moisture, potassium, sodium, iron, calcium and zinc content of *A. domesticus*. Similarly, a study in Poland on the effects of high-monosaccharide diets on development and biochemical composition of *A. domesticus* reported a significant variation in terms of fat and moisture content and non-significant variation in protein content^22^, confirming the findings of this study. Moreover their high iron content would be linked to the soil composition characterized by nitisols a soil known to be rich in iron with species sourced from territories dominated by the latter having the highest iron content^24^.

The findings is this study are consistent with the findings of Jehlík et al.^35^, who reported a significant impact of dietary behavior on biological characteristics, such as protein and fat content of *A. mellifera*. Given that honey bee nutrition is based on the nutritional stored in the hive, with nectar and honeydew as the main source of sugars and pollen, which is the key source of proteins (24%) and fat (5%), variation in pollen availability significantly affects the health and nutritional status of *A. mellifera*^36^, so a shortage and poor quality of pollen can lead to nutritional stress with huge impact on the colony^37^. This study confirms the above findings, showing that geographical source significantly impacts the nutritional and organoleptic quality of *A. mellifera*. Given the impact of biogeochemical conditions on pollen and nutritional quality, numerous attempts have been made to use artificial supplements, not only to increase bee colony and honey production^38^. The contribution of other factors such as insect development stage that could possibly explain such variations should be investigated.

Although no significant effect of geographical sourcing area on protein, fat and ash content was observed in this study, a significant effect of the latter on potassium, sodium, magnesium, iron, calcium, zinc content and sensory quality of *G. trivittata* was observed. As the effect of geographical sourcing area on nutritional and sensory quality of *G. trivittata* species is still poorly documented, a study in Togo^39^ reported higher protein values than those presented in this study. This difference could be associated not only to agro-ecological conditions under which the species was harvested^28^, but also to the conversion factors used to determine protein content. In their study, the general nitrogen-protein conversion factor (GNPCF) of 6.25 was used to calculate protein content, indicating an over-estimation of protein content. However, Boulos et al.^20^ established a nitrogen-protein conversion factor of 5.33, which was used in this study.

To date, there little to no research assessing the impact of geographical sourcing area on the nutrient and sensory quality of *G. africana*, nor characterized its nutrient and sensory quality, except a study conducted in Zimbwabwe^40^ reporting a protein content of 22 g/100 g on dry weight basis (DWB), fat (10.8 g/100 g, DWB) and ash (12.6 g/100 g, DWB). The values are lower in comparison to the ones reported in this study. This difference could be linked to the biogeochemistry differences in both geographical sourcing area. Similarly, a study conducted on *I. oyemensis* in Kabare territory^41^ reported a protein content of 56 g/100 g with a GNPCF of 6.25, fat (20.56 g/100 g) and ash of 3.11 g/100 g on DWB. These findings are comparable to those reported in this study (Mwenga territory) and lower than those reported in Kalehe territory, thus underlining the importance of the geographical sourcing area.

In their study, examining the diet effect on the chemical composition of *L. migratoria*, Oonincx and Van der Poel^23^ noted that addition of wheat bran decreased the protein content and increased fat content of the latter. The same team reported that addition of carrots to the diet increased fat content of *L. migratoria*. They also realized that mineral concentrations of Ca, K, Mg and Na were significantly affected by diet. Concentrations of P, K, Cu and Fe were significantly different in penultimate versus adult *L. migratoria*, showing that chemical composition of the latter can be manipulated by diet. These results corroborate with those reported in this study, highlighting that geographical sourcing area impact on chemical composition and sensory quality of *L. migratoria*. While a study from Thailand^42^, reported higher protein content and lower fat content as well as similar ash content in *L. migratoria*, a study from Spain^43^ noted higher fat content and comparable ash content in the latter.

In this study, *M. subhyalinus* presented macronutrient composition similar to that presented by Kinyuru et al.^44^ in Kenya with a protein content of 39.34 g/100 g and ash (7.78 g/100 g) but with a lower fat content in comparison to the one reported in Kenya. The iron, zinc and magnesium content in *M. subhylanus* noted in this study is superior, comparable and inferior depending on mineral, to mineral content reported in previous studies, some of which reported mineral contents of 6.2-10.3 mg/100 g (iron), 4.9–13.8 mg/100 g (zinc) and 39.8 mg/100 g (magnesium) for *M. subhylanus* collected in Benin, and 8.8-9.8 mg/100 g (iron) and 12-12.9 mg/100 g (zinc) for *Macrotermes* spp collected in South Africa, and 13.9 mg/100 g (iron), 12.9 mg/100 g (zinc) and 95 mg/100 g (magnesium) for *Odontotermes* spp collected in South East Asia^45^.

Finally, macronutrient composition in *R. phoenicis* reported in this study is superior to the one noted by Mba et al.^46^ in Cameroon, who reported a fat and protein contents of 21.35 g/100 g and 8.18 g/100 g fresh weight (FW), respectively. In this same perspective, Rumpold and Schlüter^47^ reported protein, fat and ash contents varying between 10.3 and 41.69 g/100 g, 19.50–55.04 g/100 g and 1.43–5.6.06 g/100 g on DWB, respectively. Additionally, Omotoso and Adedire^48^ reported mineral contents ranging from 13.67 to 17 mg/kg (sodium), 372.5–457.5 mg/kg (potassium), 43.52-60.69 mg/kg (magnesium), 6–22.90 mg/kg (iron), 0.27–2.63 mg/kg (calcium) and 0.31–0.56 mg/kg (zinc) which are inferior or comparable to mineral profile of *R. phoenicis* observed in this study depending on mineral type.

## Materials and methods

### Ethics approval

All experimental protocols, as well as methods, were approved and carried out as per relevant guidelines and regulations from the Interdisciplinary Centre for Ethical Research (CIRE) established by the Université Evangélique en Afrique, Bukavu, D.R. Congo, with reference (UEA/SGAC/KM 132/2016). The informed consent describing the study purpose was clearly explained before being signed by all subjects and/or their legal guardian (s).

### Geographical sourcing areas

**Fig. 4:**
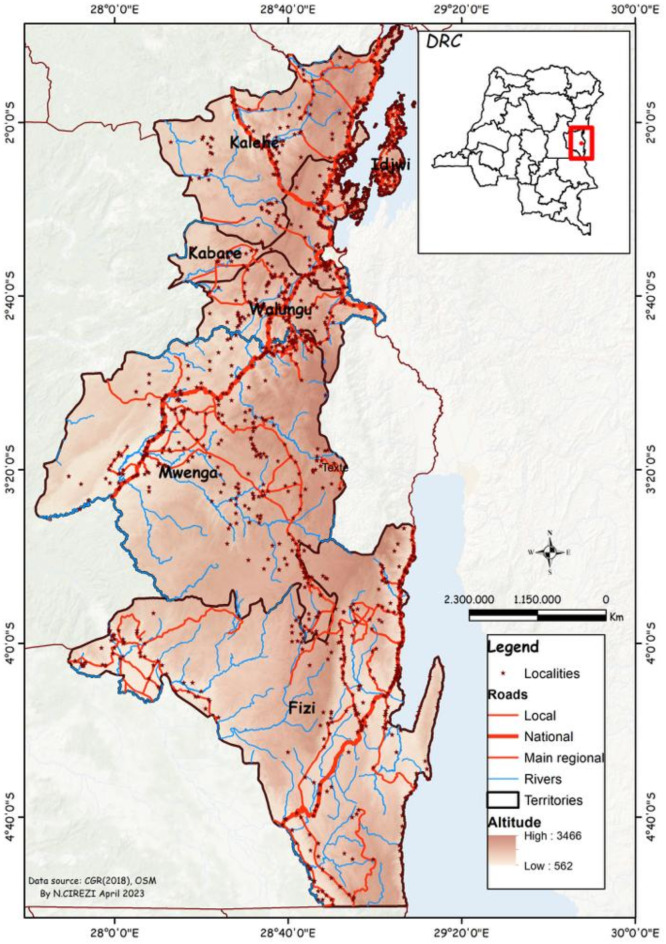
Map showing the Democratic Republic of the Congo, as well as the South-Kivu Province, and the study area (ArcMap 10.4. https://desktop.arcgis.com/en/arcmap/10.4/).

Commonly edible insect samples were obtained from six geographical areas namely Fizi, Idjwi, Kabare, Kalehe, Mwenga and Walungu, in Eastern Democratic Republic of Congo as mapped in Fig. 4.

### Agro-ecological conditions of the study area

**Table 3:** Agro-ecological conditions of the study area (retrieved from CAID/ DIAU and Inogwabini^24^.

Characteristics	Territory						
	Fizi	Walungu	Kabare		Mwenga	Kalehe	Idjwi
Latitude (South)	3° 30 to 4° 51′32	2°38′	2°30′		3° to 4°	1°37′8.85"S and 2°29′5.82"S	1°55′35.13"S and 2°16′55.40"S
Longitude (East)	27° 45 to 29° 14′10	28°40′	28°30′		28°25′29′′	29° 5′24.23"E and 28°34′15.91"E	29° 6′′′11.65"E and 29° 1′28.48"E
Area (km^2^)	15,789	1800	1960		11,172	4197	280.45
Altitude (m)	750 to 1700	1000 to 2000	1420 to 3,200		670 to 1800	788 to 3035	1439 to 2233
Climate type	Humid wet and dry tropical	Humid wet tropical	Humid wet tropical		Equatorial	Humid wet tropical	Humid wet tropical
Dominant soil unity	Acrisols and Cambisols	Ferralsols, Cambisols and Nitisols	Ferralsols and Nitisols		Acrisols and Cambisols	Haplic Acrisols, Dystric Cambisols and Haplic Nitisols, Humid Ferralsols	Gleyic Solonchaks, nitisols and Humid Ferralsols
Mean T (°C)	23.54	17–20	22.6		21–37	18–22 °C	17–30 °C
Mean Annual P(mm)	1704	900 to 1500	1572		1650	1300-2000	1,540
Estimated Population (2019)	1,093,926	1,509,175	868,616		843,636	933,181	320,009
DP (Hab.Km^− 2^)	69.3	838.4	443.6		75.5	184,6	1032,3
AEZ*	Low and high altitude	Medium to high altitude	Medium to high altitude		Low and high altitude	Low, Medium to High,	Medium to High

P (mm): Precipitation (rainfall); AEZ: Agro-Ecological Zone (High, Medium, Low); DP: Density of population; DIAU: Development Indicators Analysis Unit.

The agro-ecological conditions of the study area are depicted in Table 3. The Fizi Territory is located between 3°30 and 4°51 32 latitude (South), and 27°45 and 29°14 10 longitude (East), covering an area of ~ 15.789 km^2^ with estimated population of ~ 1.093.926 in 2019. Its elevation is subdivided into four zones, including the coastline (~ 750 m), the low land valley (~ 1000 m), a highland (~ 1300 m), and the very highland (locally called Haut Plateau with 1,700 m). The climate in Fizi is highly affected by the elevation. The rainfalls are unevenly distributed according to the month and the climatic subdivision. The North, dominated by the coastline and low inland valley, is characterized by humid tropical climate (of Aw3 type according to Köppen-Geiger classification). The greatest rainfall amounts are recorded in March and November, while the smallest amounts are the smallest amounts in February and September. The south part has a dry humid tropical climate. Available climate data mentioned an average annual rainfall of ~ 1,704 mm, the mean temperature ~ 23.54 °C (with the highest observed in April with ~ 25.6 °C and the lowest ~ 21.3 °C in September). The Territory is dominated by forest, comprising two forest reserves and a nature reserve. Acrisols and Cambisols are the dominant soil unities according to the WRB classification.

The Kabare Territory is located between 2°30′ of South latitude and 28°30’ of East longitude. Its altitude varies from ~ 1420 to 3200 m, and the Territory occupies an area of ~ 1690 km^2^ with an estimated population of ~ 868,616, which makes it among the most populated in the South-Kivu province. The Territory is located in the medium to high altitude AEZ. Available meteorological data mentioned an annual rainfall average of ~ 1572 mm, and a temperature of ~ 22.6 °C. Most of Kabare is savanna with natural vegetation consisting of wild grasses.

The Mwenga Territory is located in the middle of the province and is the only Territory surrounded by the other without any country or province borders. It is located between 28°25’29’’ East longitude and 30°02,16’05’’ South latitude. Its altitude varies between 1500 and 1800 m in the northeast. In the centre and the South, it is more or less 670 m. In the East, it is more or less 200 m and in the West more or less 670 m. It has a humid tropical climate with two seasons: the dry season from June to September and the rainy season from September to May. The temperature varies between 21 and 37 °C in most of the Territory and is low in the Itombwe area because of the high altitude, which goes up to over 2000 m. Rainfall reaches 2000 mm to 3000 mm per year. The vegetation is mainly dense forest and savanna. The forest is home to the Itombwe Nature Reserve (RNI). Relief is dominated by the Itombwe mount uplands and the alluvial valley of the Elila watershed. Soils dominated with clayey (Humic Cambisols) and sandy soil (Acrisols) types.

The Walungu Territory is located between 2º38’ of South latitude and 28º40’ of East longitude. Its altitude varies between 1000 m and 2000 m with a cold tropical climate of low altitude. There are two seasons, the dry season (June to August) and the rainy season from September to March. Available station data presented an annual average of ~ 17–20 ºC for temperature and 900 to 1500 mm rainfall. The vegetation mainly consists of grassland, a few forest reserves of Mugaba and Mushwere and woodlands scattered throughout the Territory.

The agro-ecological conditions of the study area are presented in Table 3. The Idjwi Island is located and surrounded by Lake Kivu. It is located between 1°37’8.85"S and 2°29’5.82"S as well as 29° 5’24.23"E and 28°34’15.91"E of latitude and longitude respectively. With an altitude varying from ~ 1439 m to 2,233 m (average of ~ 1811 m) and a temperature varying from 17 to 30 °C (average ~ 26.1 °C). As a result of its location (surrounded by a lake) and topography, the climate in Idjwi is humid wet tropical and tropical savannah. It is *Aw* type according to the Köppen-Geiger classification (with an average of 1540 mm of precipitation each year).

There are two seasons, the dry season (May to August) and the rainy season from September to May. The dominant soil unities according to WRB (World Reference Base for Soil) are Gleyic Solonchaks, nitisols and Humid Ferralsols, rich in sand and clay respectively. Threatened vegetation is naturally shrubby and grassy, interspersed with secondary forests. The island is also cover by croplands dominated with coffee, banana and cassava among the others. The Idjwi Territory is among the densely populated Territory in DR Congo and the region leading to high pressure on ecosystems in the island.

Kalehe is a bordering Territory between South-Kivu and North-Kivu. Located in the northern, Kalehe is one of contrasting Territory in South-Kivu based on its topography dominated by mountain (the Mitumba) in East, its altitude varies from 788 to 3035 m dividing the Territory into three AEZ: the high altitude, medium and low altitude (in Western and Northwestern). Lake Kivu borders Kalehe Territory over a distance of ~ 86 km from north to south, opening onto the Bukavu basin. The Kalehe Territory is characterized with a Humid wet tropical climate and in some area temperate with altitude. There are two seasons, the rainy season (from September to May) and the dry season (from June to August), with a precipitation ranging from 1300 to 2000 mm each year, and an annual temperature varying between 18 and 22ºC.

A diversity of soil is observed in the Kalehe Territory, from Haplic Acrisols, Dystric Cambisols, Haplic Nitisols, and Humid Ferralsols. The Dystric Cambisols and Haplic Nitisols are rich in clay very appropriate for agricultural purposes. Its vegetation is dominated by forest, where bamboos and shrubs are unfortunately in the process of disappearing due to an intense deforestation resulting in scarcity of arable land and no appropriate exploitation. Some tea, coffee, banana and cassava exploited lands are also observed. Other men activities such as small-scale mining, sand mining and livestock are dominant activities.

### Sampling and sample preparation

About 5 kg of each commonly edible insect namely *Apis mellifera* larvae, *Acheta domesticus*, *Gnathocera trivittata*, *Imbrasia oyemensis*, *Locusta migratoria*,* Gryllotalpa africana*, *Nomadacris septemfasciata*, *Macrotermes subhylanus* and *Rhyncophorus phoenicis* were collected from six geographical sources purposely selected for their familiarity with anthropo-entomophagy practices and unique agroecological conditions. Edible insect samples from each geographical sourcing area were collected using local methods as described by Ishara et al.^49,50^, then packed in zipping polyethylene bags and delivered to Université Evangelique en Afrique on flaked ice in a cool box before being washed and drained. About half of the samples were frozen at − 20 °C until further analyses and the other half was directly used for sensory assessment purposes.

### Macronutrient composition and energy

Macronutrient composition was determined in accordance with Association of Official Analytical Chemists^51^. Moisture and ash were determined by the hot-air circulating oven (105 °C) and through incineration in a muffle furnace (600 °C) respectively. Crude protein was determined by the Kjeldahl method and its content was obtained by multiplying the corresponding total nitrogen content by a factor of 5.33^20^. All determinations were carried in triplicate and expressed as mean ± standard error.

### Mineral composition

Potassium, Sodium, Magnesium, Iron, Calcium and Zinc was determined in accordance with Association of Official Analytical Chemists^51^. The mineral content was determined using AA-7000 Atomic Absorption Spectrophotometer (AAS). The residue of ashed samples was dissolved with HCl then filtered using a Whatman filter paper. The absorbance of sample and standard solutions was determined. All the analyses were performed in triplicate and expressed as mean ± standard errors.

### Sensory assessment

Insects were cooked using the methods described by Ishara et al.^34,54]^ as shown in Table 4. *A. domesticus* and *N. septemfasciata* were deep-fried for 7 min, *A. mellifera*, *I. oyemensis* and *R. phoenicis* were boiled, roasted and deep fried for 10 min, *G. trivittata* and *G. africana* were deep-fried for 10 min. Finally, *M. subhyalinus* was fried for 5 min. Sensory testing of cooked edible insects was carried out using a 7-point hedonic scale. Forty untrained panellist members from the Université Evangélique en Afrique (UEA) took part in the sensory evaluation, and the tests were carried out shortly after cooking. Samples were labelled with a random three-digit number. Between sample tests, panellists used neutral non-carbonated mineral water to rinse the palate. The evaluation was carried out at room temperature and under air circulation. Cooked edible insects were placed on a small plastic plate and a sensory evaluation in relation to appearance, aroma, taste, texture and overall score was carried out with an intensity-based questionnaire using a 7-point hedonic scale (1 = dislike extremely, 2 = dislike moderately, 3 = dislike slightly, 4 = neither like or dislike, 5 = like slightly, 6 = like moderately and 7 = like extremely) according to^52^. The geographical sourcing area and amount of ingredients used for cooking samples are briefly described in Table 4.

**Table 4:** Geographical sourcing area and amount of ingredients used for cooking samples for sensory assessment.

Insects	Water (cl)	Salt (g)	Oil (cl)	Cooking time (min)	Geographical source
*Acheta* *domesticus*	0	5	5	7	Fizi, Kabare, Mwenga & Walungu
*Apis* *mellifera*	10	5	0	10	Idjwi, Kabare, Kalehe & Walungu
*Gnathocera* *trivittata*	0	5	5	10	Kabare & Walungu
*Grillotalpa* *africana*	0	5	5	10	Kabare & Walungu
*Imbrasia* *oyemensis*	0	2	2	10	Kalehe & Mwenga
*Locusta* *migratoria*	0	5	5	7	Idjwi, Kabare, Kalehe & Walungu
*Macrotermes* *subhyalinus*	0	5	2	5	Fizi, Kabare & Walungu
*Nomadacris septemfasciata*	0	5	5	7	Kabare & Walungu
*Rhynchophorus* *phoenicis*	0	4	2	10	Fizi & Idjwi

### Statistical analysis

Data collected in triplicates were encoded in Microsoft Excel for Mac (Version 16.74). R-Studio Version 4.2.0 and Statistix Version 10 Software were used for statistical analysis, and data were presented as mean ± standard error. Analysis of variance (ANOVA) was used to delineate the effect of geographical sourcing area on the nutritional composition and sensory attributes of commonly edible insects. Means were separated using Tukey’s test at a significance level of 0.05. Cluster analysis using a non-metric multidimensional scale (NMDS) was used to determine the extent to which geographical sourcing area of origin influenced nutritional composition and sensory attributes, as well as all parameters combined. The NMDS was composed using the R package “Vegan”.

## Electronic supplementary material

Below is the link to the electronic supplementary material.

Supplementary Material 1

## Data Availability

The data supporting the findings reported herein are available on reasonable request from the corresponding author.
