# Supplementary material for: Insights into the effects of geographical sourcing area on nutrient composition and sensory attributes of nine edible insects
Source: Sci Rep. 2025 Apr 4;15:11610. doi: 10.1038/s41598-025-90659-z (PMC11971241; doi:10.1038/s41598-025-90659-z)
Supplement: Supplementary file 1 — Supplementary Material 1 [file 41598_2025_90659_MOESM1_ESM.docx]

**Supplementary information**

| Table S1. Geographical sourcing effect on macronutrient composition of commonly consumed edible insects in Eastern D. R. Congo | | | | |
| --- | --- | --- | --- | --- |
| Insect species/Territory | **Protein (g/100g)** | **Fat (g/100g)** | **Ash (g/100g)** | **MC (%)** |
| *Acheta domesticus* |  |  |  |  |
| Fizi | 37.58±1.56a | 24.00±2.00a | 11.83±0.61a | 57.23±1.57b |
| Kabare | 37.19±4.58a | 20.99±0.79b | 5.09±0.16b | 65.50±1.80a |
| Mwenga | 35.25±0.92a | 22.70±1.00ab | 4.90±1.00b | 57.74±1.02b |
| Walungu | 38.18±4.33a | 21.43±0.78b | 4.94±0.61b | 66.25±1.21a |
| p-value | 0.628 | 0.028 | <0.001 | <0.001 |
| *Apis mellifera* |  |  |  |  |
| Idjwi | 19.07±1.10a | 24.49±0.87ab | 8.79±3.92a | 70.06±1.02b |
| Kabare | 20.08±0.49a | 24.38±1.16ab | 4.72±0.36b | 71.23±2.08b |
| Kalehe | 19.66±2.51a | 26.29±1.72a | 4.89±0.31b | 78.84±2.11a |
| Walungu | 19.82±1.02a | 22.27±0.91b | 4.41±0.59b | 71.59±1.72b |
| p-value | 0.793 | 0.023 | 0.043 | <0.001 |
| *Gnathocera trivittata* |  |  |  |  |
| Kabare | 34.40±0.66a | 17.78±0.29a | 4.94±0.61a | 62.21±1.68a |
| Walungu | 36.13±2.92a | 16.90±0.7a | 6.57±0.86a | 56.40±1.78b |
| p-value | 0.305 | 0.115 | 0.055 | 0.015 |
| *Grillotalpa africana* |  |  |  |  |
| Kabare | 30.70±1.10a | 26.73±0.63a | 5.26±0.46a | 63.66±0.35b |
| Walungu | 32.22±1.25a | 21.23±0.96b | 4.45±0.33a | 79.72±1.45a |
| p-value | 0.136 | <0.001 | 0.067 | <0.001 |
| *Imbrasia oyemensis* |  |  |  |  |
| Kalehe | 31.18±1.09b | 27.95±1.41a | 6.93±0.18a | 79.05±2.30a |
| Mwenga | 56.68±1.20a | 15.68±1.00b | 5.92±1.01a | 66.58±1.07b |
| p-value | <0.001 | <0.001 | 0.166 | <0.001 |
| *Locusta migratoria* |  |  |  |  |
| Idjwi | 29.63±0.90b | 21.97±0.15ab | 5.17±1.02a | 69.03±1.60b |
| Kabare | 35.25±1.53a | 21.14±0.25ab | 5.67±0.50a | 59.82±0.22c |
| Kalehe | 30.08±0.36b | 16.95±0.73c | 5.56±0.68a | 77.34±2.32a |
| Walungu | 30.22±1.08b | 23.10±1.51a | 4.54±1.17a | 69.22±1.46b |
| p-value | <0.001 | <0.001 | 0.437 | <0.001 |
| *Macrotermes subhyalinus* |  |  |  |  |
| Fizi | 29.00±1.06a | 27.10±0.93a | 8.93±0.68a | 45.47±0.55c |
| Kabare | 25.77±2.05b | 26.34±1.59a | 5.01±0.39b | 70.33±0.59b |
| Walungu | 27.88±2.19ab | 24.90±1.23b | 4.55±0.52b | 77.27±2.85a |
| p-value | 0.011 | 0.021 | <0.001 | <0.001 |
| *Nomadacris septemfasciata* |  |  |  |  |
| Kabare | 24.54±1.57a | 38.28±1.53a | 5.50±0.82a | 56.43±1.16b |
| Walungu | 30.61±4.86a | 11.97±1.29b | 4.30±0.16a | 66.31±0.84a |
| p-value | 0.073 | <0.001 | 0.069 | <0.001 |
| *Rhynchophorus phoenicis* |  |  |  |  |
| Fizi | 31.55±0.56b | 25.80±1.31b | 6.92±1.62a | 68.84±0.56a |
| Idjwi | 39.19±3.44a | 30.60±1.05a | 7.02±0.69a | 68.14±0.32a |
| p-value | 0.011 | 0.008 | 0.924 | 0.133 |

Mean values (n=3)±SE. All values except moisture are expressed on dry weight.

Values in the same column with the same following letter do not significantly differ (p<0.05).

MC: Moisture content.

| Table S2. Geographical sourcing effect on mineral profile (mg/100g) of commonly consumed edible insects in Eastern D. R. Congo | | | | | | |
| --- | --- | --- | --- | --- | --- | --- |
| Insect species/Territory | **Potassium** | **Sodium** | **Magnesium** | **Iron** | **Calcium** | **Zinc** |
| *Acheta domesticus* |  |  |  |  |  |  |
| Fizi | 148.25±0.49a | 160.67±0,81a | 47.60±0.60a | 6.60±0.20a | 144.27±4.56a | 15.85±0.35a |
| Kabare | 144.56±1.56a | 146.00±1.00c | 46.60±9.00a | 6.27±0.23a | 143.00±4.00a | 14.50±0.10b |
| Mwenga | 67.27±3.88c | 157.40±1.00b | 59.60±20.00a | 4.10±0.10c | 131.00±1.00b | 12.20±1.00c |
| Walungu | 110.93±0.67b | 161.23±0.93a | 40.90±0.56a | 5.87±0.29b | 87.00±1.65c | 15.40±0.36ab |
| p-value | <0.001 | <0.001 | 0.280 | <0.001 | <0.001 | <0.001 |
| *Apis mellifera* |  |  |  |  |  |  |
| Idjwi | 98.37±1.37ab | 143.50±1.42d | 49.27±6.33a | 9.24±0.06a | 147.40±0.56a | 14.68±0.63a |
| Kabare | 94.20±3.13b | 154.70±0.40b | 50.10±2.90a | 6.60±0.20b | 124.00±1.00b | 15.00±0.60a |
| Kalehe | 101.70±3.54a | 152.23±1.24c | 51.72±3.97a | 6.50±0.85b | 124.33±2.52b | 14.53±0.72a |
| Walungu | 74.79±1.94c | 158.67±1.53a | 45.33±0.78a | 4.87±0.21c | 126.33±1.53b | 14.27±0.47a |
| p-value | <0.001 | <0.001 | 0.321 | <0.001 | <0.001 | 0.399 |
| *Gnathocera trivittata* |  |  |  |  |  |  |
| Kabare | 19.54±0.63a | 161.30±0.30a | 32.40±2.50b | 8.80±0.10a | 128.50±7.50b | 14.40±0.50a |
| Walungu | 105.38±1.20b | 144.23±1.07b | 54.77±1.30a | 6.50±0.53b | 148.33±3.21a | 15.83±0.90a |
| p-value | <0.001 | <0.001 | <0.001 | <0.001 | 0.013 | 0.496 |
| *Grillotalpa africana* |  |  |  |  |  |  |
| Kabare | 46.91±1.41b | 168.00±0.20a | 24.30±0.34b | 5.09±0.21a | 129.50±0.50b | 15.10±0.40a |
| Walungu | 81.03±1.03a | 141.47±0.72b | 42.17±1.21a | 5.53±0.38a | 162.00±2.00a | 16.30±0.44a |
| p-value | <0.001 | <0.001 | <0.001 | 0.373 | <0.001 | 0.101 |
| *Imbrasia oyemensis* |  |  |  |  |  |  |
| Kalehe | 193.16±2.15a | 159.23±0.88a | 66.96±9.08a | 7.47±0.49a | 107.33±1.53a | 14.36±1.63a |
| Mwenga | 56.43±1.19b | 154.44±1.16b | 60.57±0.86a | 7.57±0.90a | 102.00±1.00b | 12.38±1.22a |
| p-value | <0.001 | 0.004 | 0.306 | 0.643 | 0.007 | 0.158 |
| *Locusta migratoria* |  |  |  |  |  |  |
| Idjwi | 73.43±0.97d | 157.18±1.02b | 56.00±4.40b | 4.15±0.05b | 131.49±0.50b | 13.31±0.38b |
| Kabare | 77.75±1.26c | 154.00±0.56c | 51.40±1.10bc | 6.00±0.10a | 153.00±4.00a | 13.10±0.80b |
| Kalehe | 87.40±2.17b | 157.54±1.51b | 63.43±6.26a | 5.93±0.40a | 125.33±1.53c | 13.19±1.37b |
| Walungu | 108.05±1.27a | 162.90±0.66a | 44.83±0.60c | 7.17±0.64a | 122.67±2.52c | 17.13±0.35a |
| p-value | <0.001 | <0.001 | 0.002 | 0.003 | <0.001 | <0.001 |
| *Macrotermes subhyalinus* |  |  |  |  |  |  |
| Fizi | 480.47±0.39b | 157.67±0.58b | 28.73±1.12b | 5.57±0.42a | 139.10±0.85a | 17.57±0.75a |
| Kabare | 160.43±0.03c | 163.90±0.40a | 20.60±1.60c | 5.90±0.23a | 135.50±0.50b | 17.80±0.40a |
| Walungu | 520.44±19.77a | 164.33±0.68a | 67.87±0.97a | 5.20±0.20a | 97.33±1.53c | 15.10±0.36b |
| p-value | <0.001 | <0.001 | <0.001 | 0.123 | <0.001 | 0.009 |
| *Nomadacris septemfasciata* |  |  |  |  |  |  |
| Kabare | 54.37±2.15b | 162.70±1.40a | 29.80±6.50b | 6.80±0.20a | 160.50±2.50a | 13.20±0.30a |
| Walungu | 116.71±0.77a | 162.07±1.69a | 56.27±1.16a | 7.79±0.36a | 153.00±1.00b | 13.03±0.70a |
| p-value | <0.001 | 0.621 | 0.002 | 0.101 | 0.009 | 0.865 |
| *Rhynchophorus phoenicis* |  |  |  |  |  |  |
| Fizi | 28.93±0.73a | 169.33±0.58a | 32.30±0.30b | 8.57±0.42a | 176.05±0.45a | 18.30±0.60a |
| Idjwi | 27.65±0.76a | 170.67±1.35a | 58.93±4.46a | 5.43±0.81b | 98.97±0.35b | 13.00±2.05b |
| p-value | 0.101 | 0.349 | <0.001 | <0.001 | <0.001 | 0.029 |

Mean values (n=3)±SE on wet basis. Values in the same column with the same following letter do not significantly differ (p<0.05).

| Table S3. Geographical sourcing effect on sensory attributes of commonly consumed edible insects in Eastern D. R. Congo | | | | | | |
| --- | --- | --- | --- | --- | --- | --- |
| Insect species/Territory | **Appearance** | **Aroma** | **Texture** | **Taste** | **After taste** | **Overall acceptability** |
| *Acheta domesticus* |  |  |  |  |  |  |
| Fizi | 3.90±0.84a | 4.53±0.91a | 4.03±0.80a | 3.85±0.77a | 3.85±0.74a | 4.03±0.52a |
| Kabare | 3.93±1.90a | 3.88±1.77b | 3.90±1.85a | 4.23±1.56a | 4.15±1.46a | 4.02±0.66a |
| Mwenga | 3.78±0.97a | 4.85±0.83a | 4.05±0.81a | 3.83±0.96a | 3.88±1.07a | 4.08±0.68a |
| Walungu | 3.75±0.84a | 4.75±0.74a | 4.08±0.76a | 3.75±0.93a | 3.70±0.85a | 4.01±0.60a |
| p-value | 0.892 | <0.001 | 0.909 | 0.215 | 0.297 | 0.896 |
| *Apis mellifera* |  |  |  |  |  |  |
| Idjwi | 4.78±0.92ab | 4.50±0.99ab | 4.35±0.92a | 5.70±0.72a | 5.20±0.69a | 4.91±0.42a |
| Kabare | 4.30±1.99b | 3.95±1.89b | 3.98±1.42a | 4.23±1.76bc | 4.48±1.36b | 4.19±0.86b |
| Kalehe | 4.58±1.06ab | 4.73±1.13a | 4.48±1.30a | 4.73±1.28b | 4.75±1.33ab | 4.65±0.58a |
| Walungu | 5.10±1.17a | 4.40±1.06ab | 3.23±1.03b | 3.93±0.89c | 3.53±1.15c | 4.04±0.71b |
| p-value | 0.036 | 0.047 | <0.001 | <0.001 | < 0.001 | <0.001 |
| *Gnathocera trivittata* |  |  |  |  |  |  |
| Kabare | 3.75±1.79a | 4.33±1.62b | 3.50±1.30b | 4.23±1.49a | 4.40±1.81a | 4.04±0.81a |
| Walungu | 2.95±1.08b | 5.00±0.93a | 4.28±0.78a | 4.35±0.83a | 3.93±0.86a | 4.10±0.50a |
| p-value | 0.018 | 0.025 | 0.002 | 0.645 | 0.138 | 0.547 |
| *Grillotalpa africana* |  |  |  |  |  |  |
| Kabare | 3.48±1.48b | 4.03±1.48b | 4.70±1.02b | 4.68±1.31b | 4.50±1.15a | 4.28±0.50b |
| Walungu | 4.13±0.76a | 5.40±0.74a | 5.23±0.73a | 5.30±0.72a | 4.88±0.76a | 4.99±0.46a |
| p-value | 0.016 | <0.001 | <0.001 | <0.001 | 0.090 | <0.001 |
| *Imbrasia oyemensis* |  |  |  |  |  |  |
| Kalehe | 3.83±1.62a | 3.95±1.20b | 4.08±1.19b | 4.25±1.63a | 3.93±1.94b | 4.01±0.81b |
| Mwenga | 3.73±1.47a | 4.65±1.29a | 4.78±1.12a | 4.88±1.28a | 4.90±1.06a | 4.61±0.43a |
| p-value | 0.867 | 0.014 | 0.008 | 0.060 | 0.007 | <0.001 |
| *Locusta migratoria* |  |  |  |  |  |  |
| Idjwi | 4.88±1.04ab | 5.20±0.99b | 5.00±1.04a | 5.35±0.95ab | 4.60±0.74b | 5.01±0.50a |
| Kabare | 4.88±1.65ab | 5.20±1.32b | 4.83±1.34a | 5.13±1.11ab | 5.00±1.34ab | 5.01±0.64a |
| Kalehe | 5.03±1.19a | 5.08±1.00b | 5.10±0.98a | 5.03±1.10b | 4.83±0.90ab | 5.01±0.72a |
| Walungu | 4.48±0.93b | 5.88±0.79a | 5.25±0.74a | 5.55±0.78a | 5.08±1.02a | 5.25±0.54a |
| p-value | 0.022 | 0.003 | 0.326 | 0.047 | 0.017 | 0.431 |
| *Macrotermes subhyalinus* |  |  |  |  |  |  |
| Fizi | 5.25±0.71b | 6.23±0.77a | 5.28±0.68b | 6.30±0.82ab | 6.25±0.84a | 5.86±0.56a |
| Kabare | 5.85±1.14a | 6.15±0.83a | 5.90±0.84a | 5.93±1.00b | 6.25±0.81a | 6.02±0.43a |
| Walungu | 5.25±0.71b | 6.35±0.74a | 5.28±0.75b | 6.48±0.72a | 6.35±0.83a | 5.94±0.56a |
| p-value | 0.003 | 0.513 | <0.001 | 0.015 | 0.823 | 0.322 |
| *Nomadacris septemfasciata* |  |  |  |  |  |  |
| Kabare | 4.73±1.26a | 5.35±1.14a | 5.65±1.19a | 5.13±1.16b | 5.03±1.10a | 5.18±0.57a |
| Walungu | 4.63±0.90a | 5.68±1.12a | 5.60±0.96a | 5.75±0.78a | 5.20±1.02a | 5.37±0.61a |
| p-value | 0.684 | 0.203 | 0.834 | 0.006 | 0.462 | 0.238 |
| *Rhynchophorus phoenicis* |  |  |  |  |  |  |
| Fizi | 6.08±0.57a | 5.38±0.59a | 5.58±0.56a | 6.38±0.70a | 6.03±0.66a | 5.89±0.36a |
| Idjwi | 4.28±0.85b | 4.53±1.04b | 4.40±1.01b | 4.88±0.88b | 4.53±0.72b | 4.52±0.41b |
| p-value | <0.001 | <0.001 | <0.001 | <0.001 | <0.001 | <0.001 |

Mean values (n=40)±SE. Values in the same column with the same following letter do not significantly differ (p<0.05).
